# Supplementary material for: In-situ self-assembly of hole transport monolayer during crystallization for efficient single-crystal perovskite solar cells
Source: Nat Commun. 2025 Aug 6;16:7245. doi: 10.1038/s41467-025-62393-7 (PMC12328746; doi:10.1038/s41467-025-62393-7)
Supplement: Supplementary file 1 — Supplementary Information [file 41467_2025_62393_MOESM1_ESM.pdf]

# Supplementary Information

# In-Situ Self-Assembly of Hole Transport Monolayer During Crystallization for Efficient Single-Crystal Perovskite Solar Cells

Vishal Yeddu[1#], Khulud Almasabi[2#], Yafeng Xu[2], Augusto Amaro[1], Shuang Qiu[1], Sergey Dayneko[1], Dongyang Zhang[1], Parinaz Moazzezi[3], Christopher Tremblay[1,4], Muhammad Naufal Lintangpradipto[2], Heather L. Buckley[1,4,5], Omar F. Mohammed[2], Osman Bakr[2*], and Makhsud I. Saidaminov.[1,3,4*]

[1]Department of Chemistry, University of Victoria, 3800 Finnerty Road, Victoria, British Columbia V8P 5C2, Canada

[2]Center for Renewable Energy and Storage Technologies (CREST), Division of Physical Science and Engineering, King Abdullah University of Science and Technology, Thuwal, 23955-6900 Kingdom of Saudi Arabia

[3]Department of Electrical & Computer Engineering, University of Victoria, 3800 Finnerty Road, Victoria, British Columbia V8P 5C2, Canada

[4]Center for Advanced Materials and Related Technologies (CAMTEC), University of Victoria, Victoria, British Columbia, V8P 5C2 Canada

[5]Department of Civil Engineering, University of Victoria, 3800 Finnerty Road, Victoria, BC V8P 5C2, Canada

[#] Equal author contribution

[*]Corresponding authors

These authors contributed equally: Vishal Yeddu, Khulud Almasabi

These authors jointly supervised this work: Osman Bakr, Makhsud I. Saidaminov

Corresponding authors e-mails:

osman.bakr@kaust.edu.sa (Osman Bakr), msaidaminov@uvic.ca (Makhsud I. Saidaminov)

# Contents

**Supplementary Fig. 1: Perovskite crystal size distribution.** ........ 3
**Supplementary Fig. 2: Optical micrograph of crystal surface.** ........ 4
**Supplementary Fig. 3: Champion ASSC ITO device characteristics (J-V and EQE).** ........ 5
**Supplementary Fig. 4: Comparison of SSSC and ASSC ITO SC-PSC parameters.** ........ 6
**Supplementary Fig. 5: J-V characteristics of champion SSSC and ASSC ITO SC-PSC.** ........ 7
**Supplementary Fig. 6: Characterization of different substrate types using CV.** ........ 8
**Supplementary Fig. 7: XRD of ITO.** ........ 9
**Supplementary Fig. 8: Temperature difference between top and bottom substrate.** ........ 10
**Supplementary Fig. 9: Contact angle measurements.** ........ 11
**Supplementary Fig. 10: Characterization of surfaces using AFM and KPFM.** ........ 12
**Supplementary Fig. 11: TPC and TPV measurements of SC-PSCs.** ........ 13
**Supplementary Fig. 12: TRPL of the detached crystals.** ........ 14
**Supplementary Fig. 13: Shelf-stability of SC-PSCs.** ........ 15
**Supplementary Fig. 14: Stability of SC-PSCs under ambient conditions.** ........ 16
**Supplementary Fig. 15: Operational stability of SC-PSCs.** ........ 17

**Supplementary Figures:**

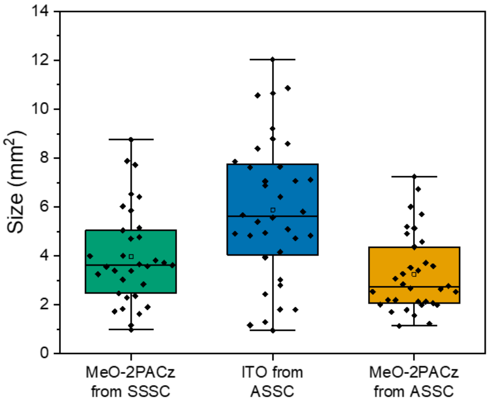


**Supplementary Fig. 1: Perovskite crystal size distribution.**

Box-and-whisker plots showing size distribution of perovskite crystals for each substrate type. The box boundaries represent the first and third quartiles (25$^{th}$ and 75$^{th}$ percentiles), the horizontal line within each box indicates the median, and the hollow square marks the average crystal size. The whiskers extending above and below each box show the range of values that fall within the normal spread.

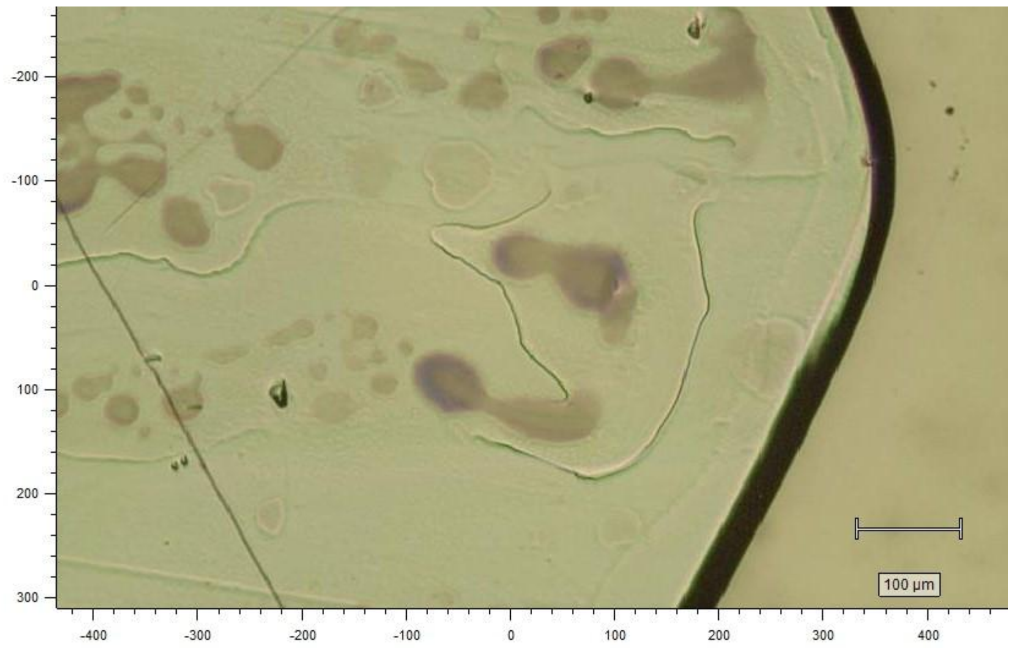


**Supplementary Fig. 2: Optical micrograph of crystal surface.**
Optical micrograph of $FA_{0.6}MA_{0.4}PbI_3$ single crystal surface zooming on spots observed in the pictures of perovskite crystals.

a

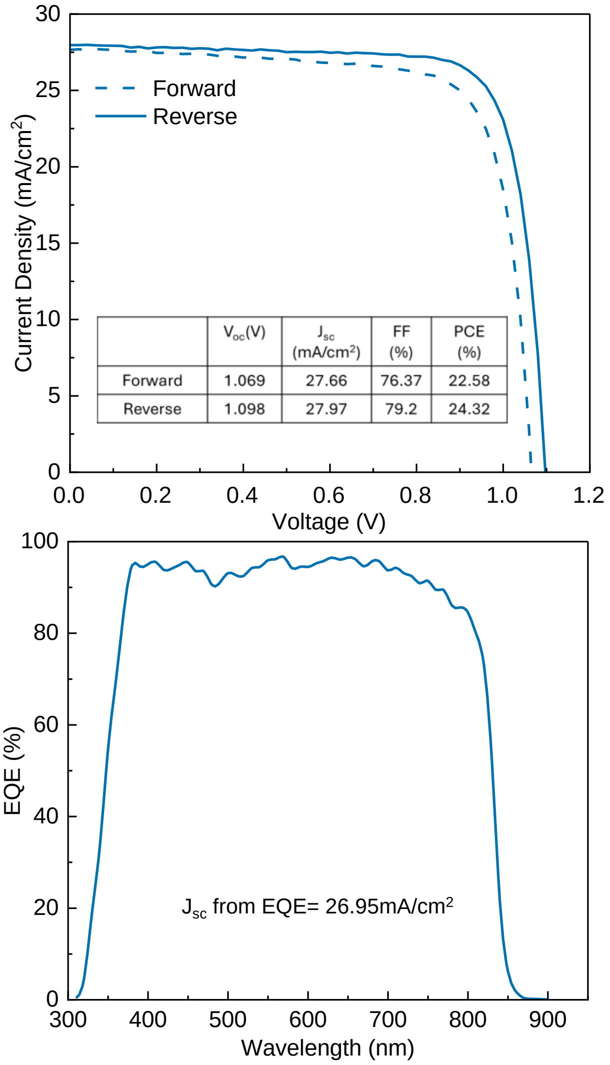


| | $V_{oc}$(V) | $J_{sc}$ (mA/cm$^2$) | FF (%) | PCE (%) |
|---|---|---|---|---|
| Forward | 1.069 | 27.66 | 76.37 | 22.58 |
| Reverse | 1.098 | 27.97 | 79.2 | 24.32 |

**Supplementary Fig. 3: Champion ASSC ITO device characteristics (J-V and EQE).**

**a** Forward and reverse J-V characteristics of the champion ASSC ITO SC-PSC. The table in the inset presents the open-circuit voltage ($V_{oc}$), short-circuit current density ($J_{sc}$), fill factor (FF), and power conversion efficiency (PCE) for both the forward and reverse scans. **b** EQE of the champion ASSC ITO SC-PSC.

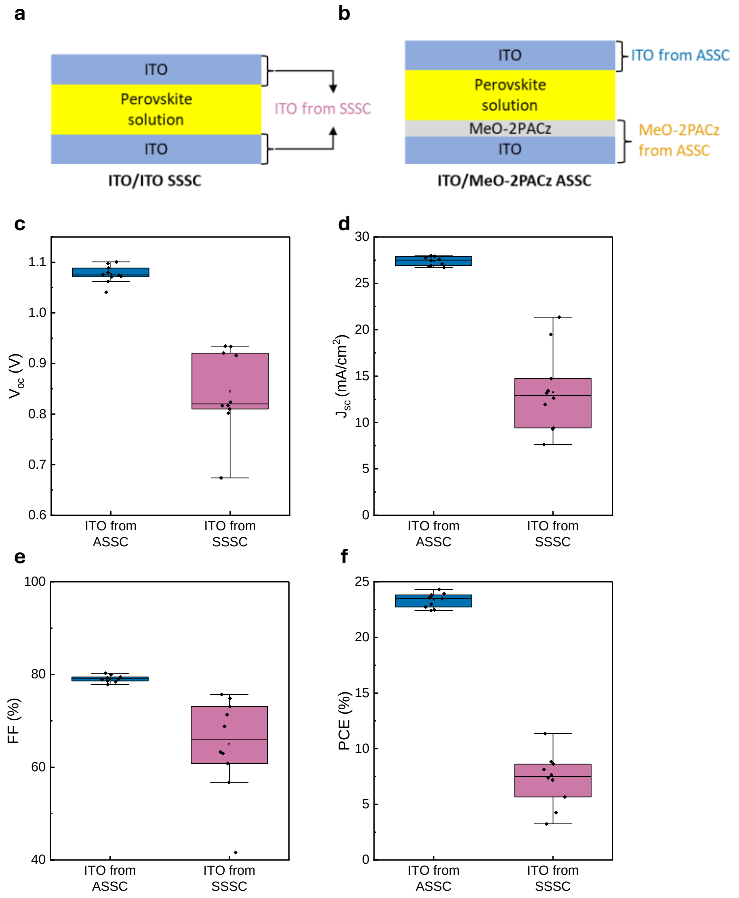


**Supplementary Fig. 4: Comparison of SSSC and ASSC ITO SC-PSC parameters.**

**a** ITO/ITO symmetric substrate stack configuration and **b** ITO/MeO-2PACz asymmetric substrate stack configuration. Statistical **c** $V_{oc}$, **d** $J_{sc}$, **e** FF **f** PCE data for 10 SC-PSCs on each type of SSSC and ASSC ITO. In the box plots, the top and bottom edges of each box indicate the first and third quartiles (the 25$^{th}$ and 75$^{th}$ percentiles). This illustrates the spread of the middle half of the data. The line inside the box is the median line, highlighting the data's central tendency. The whiskers extending above and below each box show the range of values that fall within the normal spread.

**a**

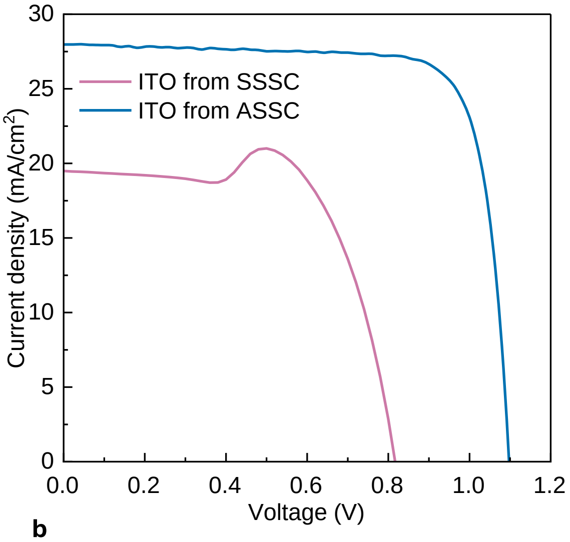


**b**

| | $V_{oc}$(V) | $J_{sc}$ (mA/cm$^2$) | FF (%) | PCE (%) |
|---|---|---|---|---|
| ITO from SSSC | 0.817 | 19.49 | 71.31 | 11.35 |
| ITO from ASSC | 1.098 | 27.97 | 79.2 | 24.32 |

**Supplementary Fig. 5: J-V characteristics of champion SSSC and ASSC ITO SC-PSC.**

**a** Reverse scan J-V characteristics of the champion SSSC ITO and ASSC ITO SC-PSCs. **b** Table summarizing the solar cell parameters namely open circuit voltage ($V_{oc}$), short circuit current density ($J_{sc}$), fill factor (FF) and power conversion efficiency (PCE) of the champion SSSC ITO and ASSC ITO SC-PSCs.

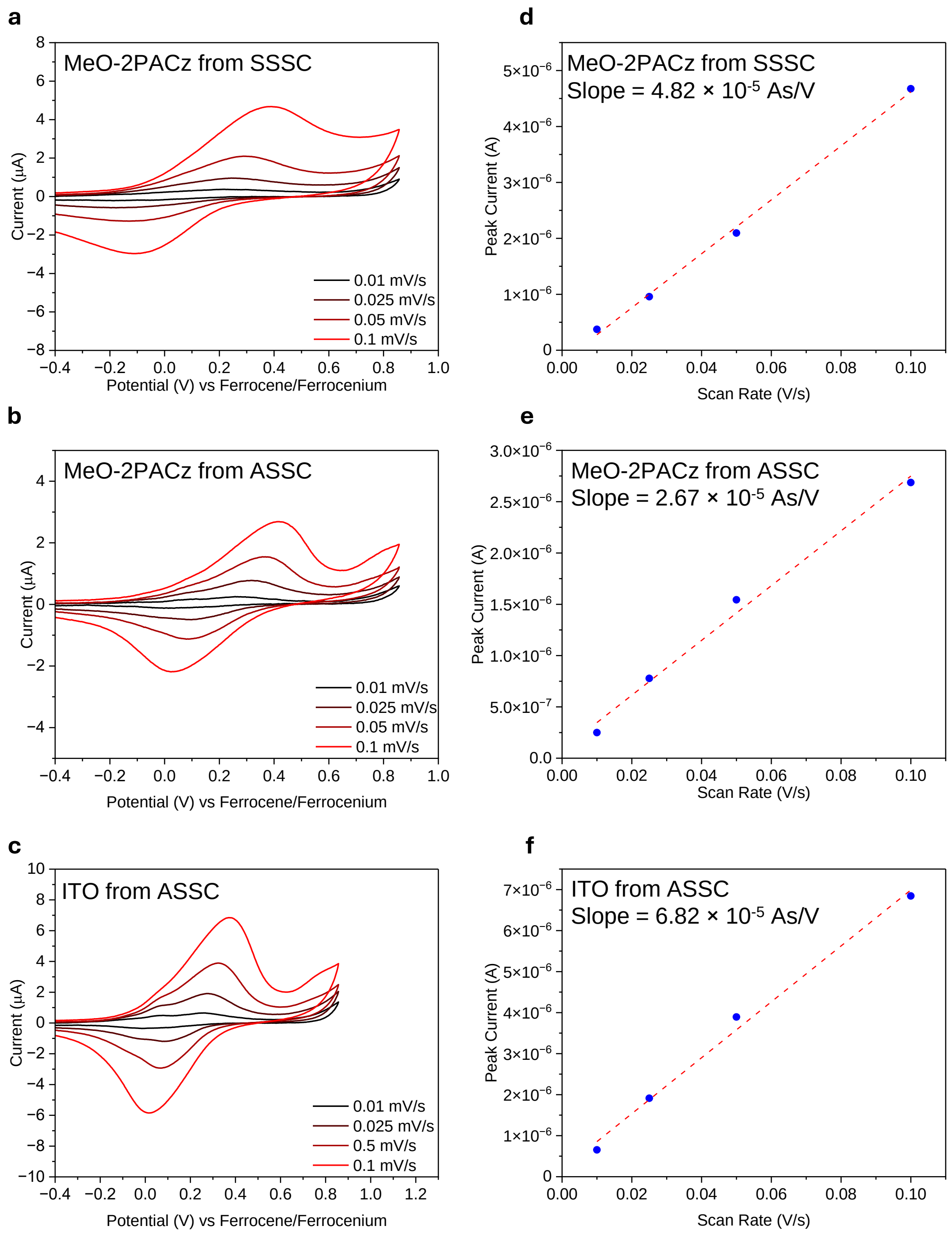


**Supplementary Fig. 6: Characterization of different substrate types using CV.**

CV curves of **a** symmetric MeO-2PACz, **b** asymmetric MeO-2PACz, and **c** asymmetric ITO, measured in oDCB solution at different scan rates. Panels **d-f** show the corresponding peak current vs. scan rate plots.

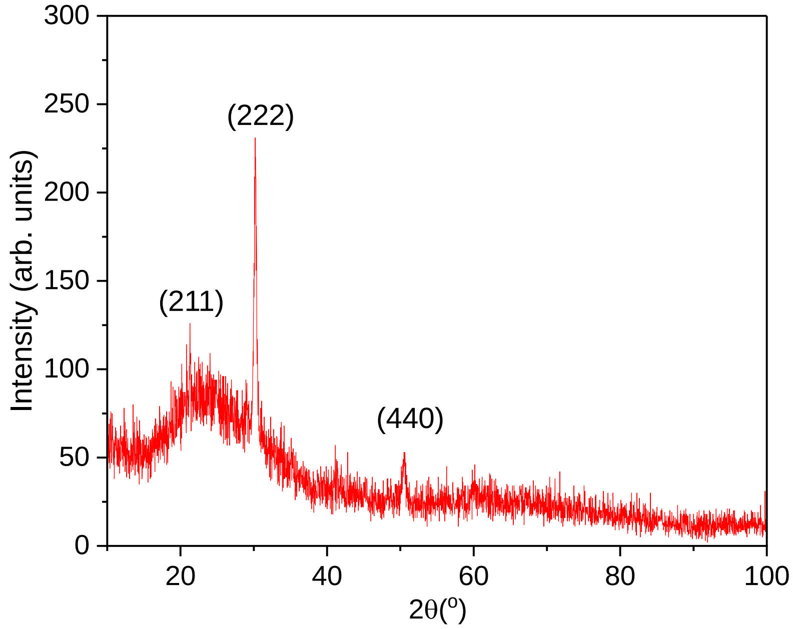


**Supplementary Fig. 7: XRD of ITO.**

XRD pattern of ITO coated glass substrate.

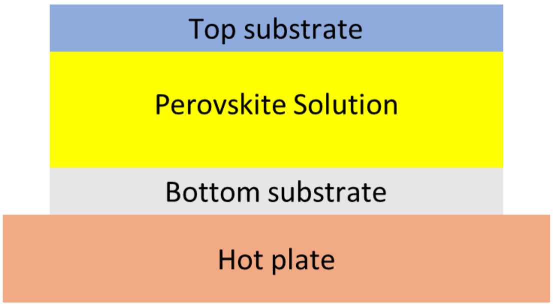


| Bottom substrate temperature (°C) | Top substrate temperature (°C) |
|---|---|
| 50 | 47 |
| 70 | 66 |
| 90 | 83 |
| 110 | 91 |
| 130 | 117 |

**Supplementary Fig. 8: Temperature difference between top and bottom substrate.**
Measured temperature of top and bottom substrates in degree Celsius (°C) during crystallization.

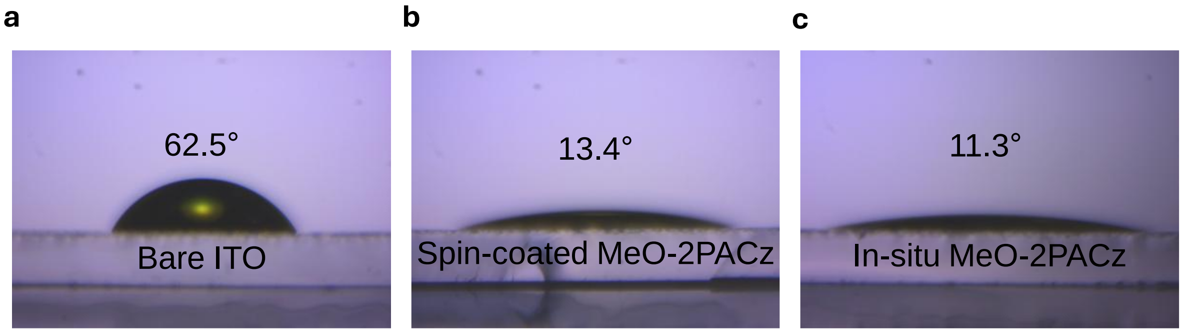


**Supplementary Fig. 9: Contact angle measurements.**

Contact angle measurement of **a** bare ITO, **b** spin-coated MeO-2PACz and **c** in-situ coated MeO-2PACz. The measurements were performed using supersaturated $FA_{0.6}MA_{0.4}PbI_3$ perovskite solution in gamma-butyrolactone (GBL).

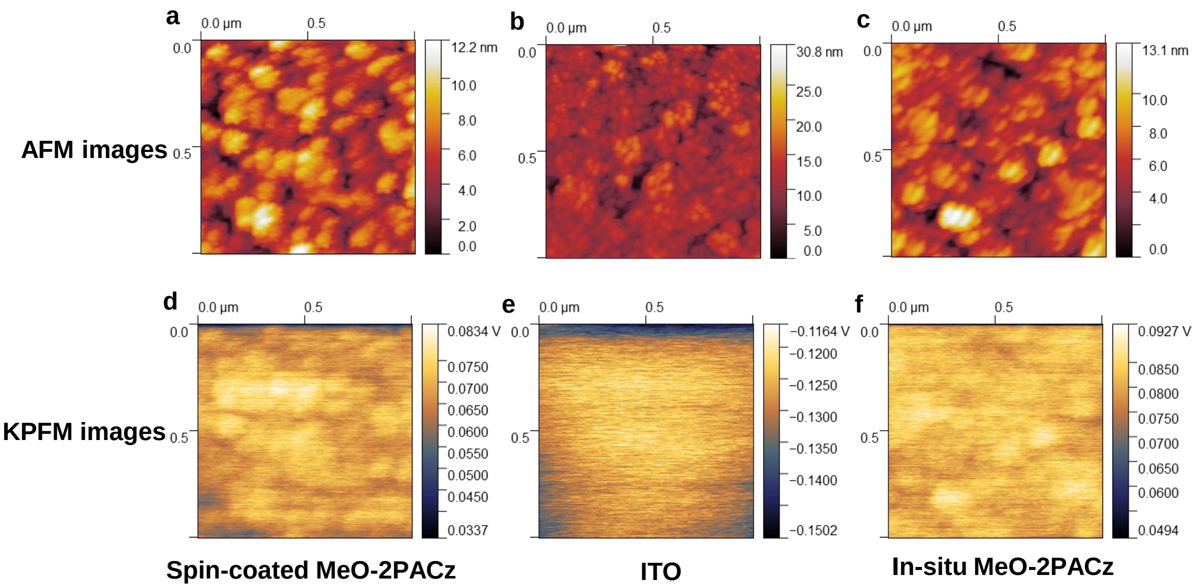


**Supplementary Fig. 10: Characterization of surfaces using AFM and KPFM.**

AFM micrographs of **a** ITO spin-coated with MeO-2PACz, **b** uncoated bare ITO and **c** ITO in-situ coated with MeO-2PACz. KPFM micrographs of **d** ITO spin-coated with MeO-2PACz, **e** uncoated bare ITO and **f** ITO in-situ coated with MeO-2PACz.

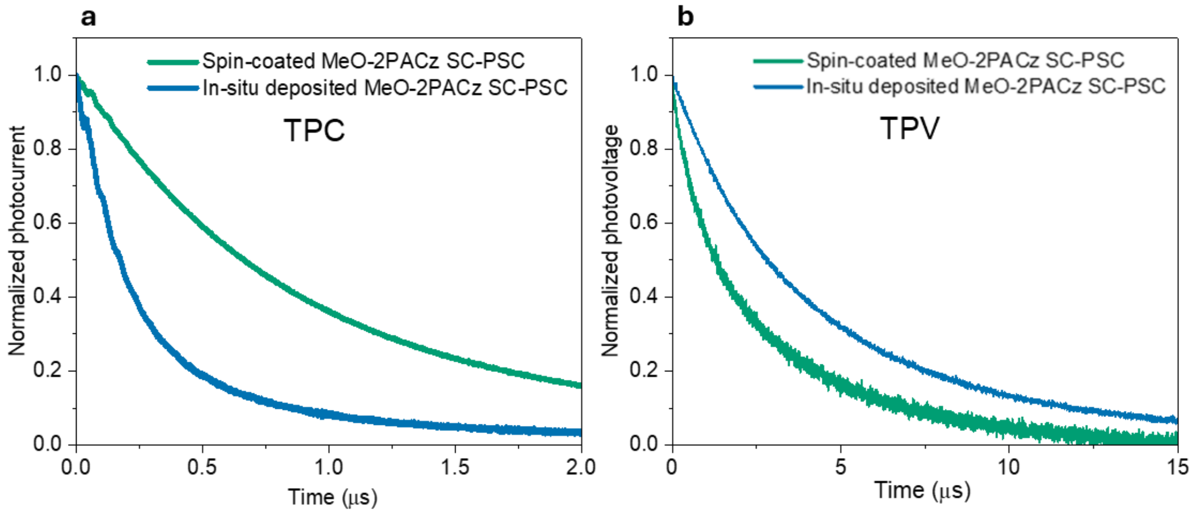


**Supplementary Fig. 11: TPC and TPV measurements of SC-PSCs.**

**a** TPC and **b** TPV of SC-PSCs with spin and in-situ deposited MeO-2PACz as hole transport layer.

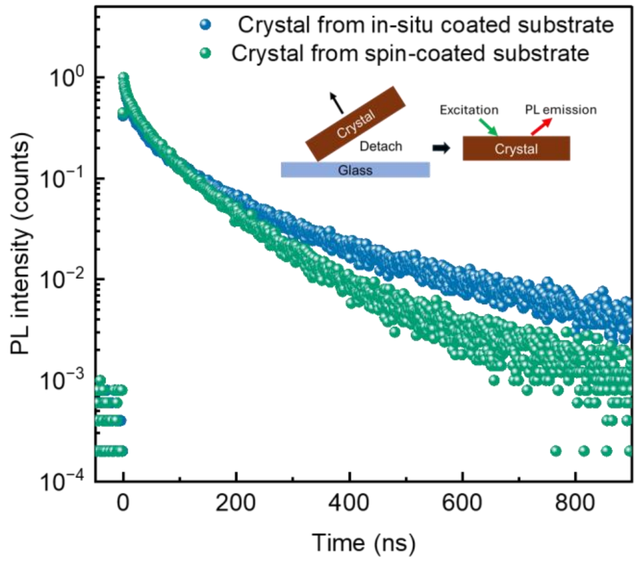


**Supplementary Fig. 12: TRPL of the detached crystals.**

TRPL of perovskite crystals which were detached from substrates having spin and in-situ coated MeO-2PACz. PL intensity counts have been normalized.

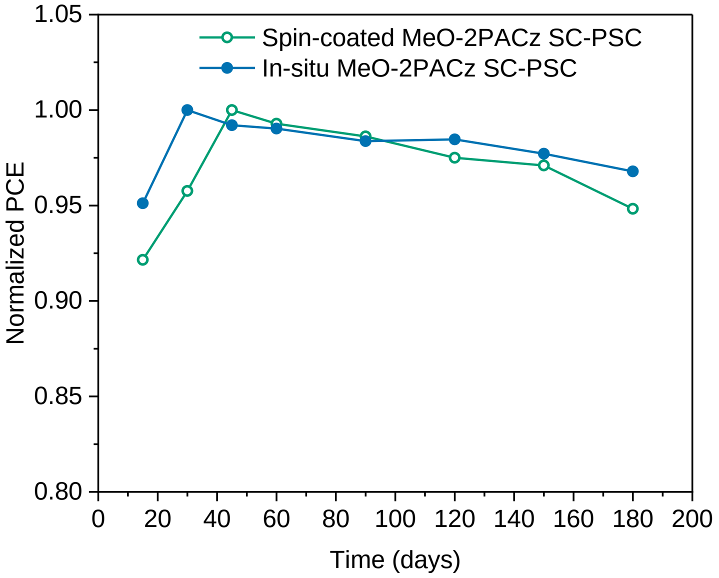


**Supplementary Fig. 13: Shelf-stability of SC-PSCs.**

Performance evolution of spin-coated MeO-2PACz SC-PSC and in-situ MeO-2PACz SC-PSC, with each device's data normalized to its respective peak PCE value rather than its initial PCE. The devices were stored in nitrogen glovebox in between measurements.

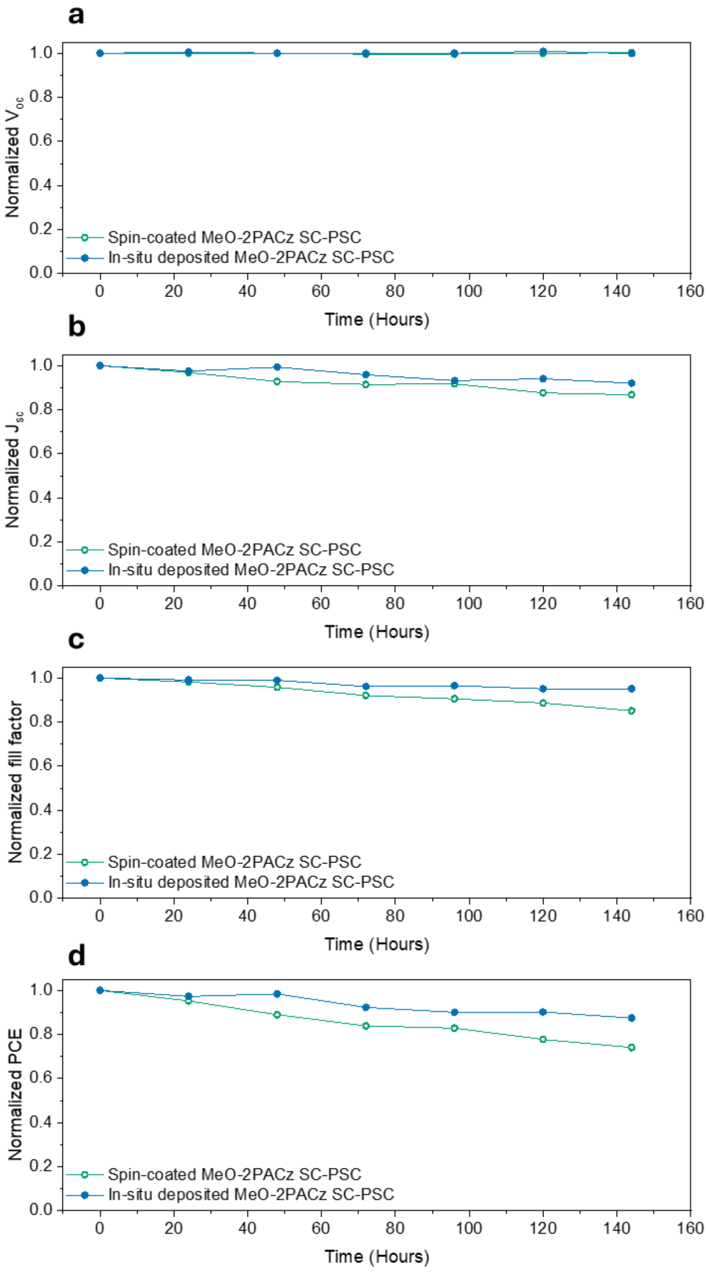


**Supplementary Fig. 14: Stability of SC-PSCs under ambient conditions.**

Evolution of (a) $V_{oc}$, (b) $J_{sc}$ (c) fill factor and (d) PCE of spin-coated and in-situ deposited SC-PSCs upon storage in ambient conditions (23 °C, 45 ± 10 % relative humidity).

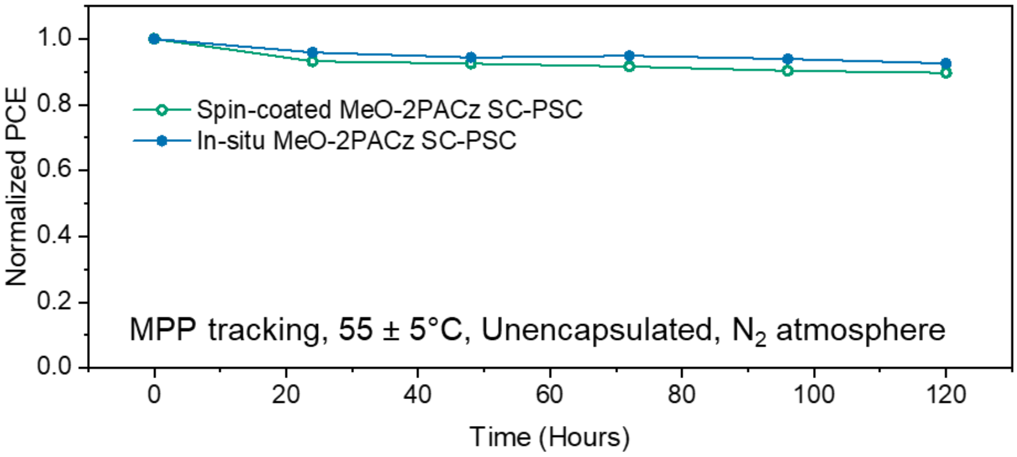


**Supplementary Fig. 15: Operational stability of SC-PSCs.**

Operational stability of spin-coated and in-situ deposited MeO-2PACz SC-PSCs determined by MPP tracking. Measurements were performed using unencapsulated SC-PSCs inside $N_2$ filled glovebox at around 55 °C.
