# Supplementary material for: In-situ self-assembly of hole transport monolayer during crystallization for efficient single-crystal perovskite solar cells
Source: Nat Commun. 2025 Aug 6;16:7245. doi: 10.1038/s41467-025-62393-7 (PMC12328746; doi:10.1038/s41467-025-62393-7)
Supplement: Supplementary file 2 — Description Of Additional Supplementary File [file 41467_2025_62393_MOESM2_ESM.pdf]

Description of additional supplementary files

Supplementary Data 1

Atomic coordinates of the optimized computational model of MeO-2PACz molecule on ITO(111) plane.

Supplementary Data 2

Crystallographic Information File of the optimized computational model of MeO-2PACz molecule on ITO(111) plane.
