# Supplementary material for: In-situ self-assembly of hole transport monolayer during crystallization for efficient single-crystal perovskite solar cells
Source: Nat Commun. 2025 Aug 6;16:7245. doi: 10.1038/s41467-025-62393-7 (PMC12328746; doi:10.1038/s41467-025-62393-7)
Supplement: Supplementary file 5 — Reporting Summary [file 41467_2025_62393_MOESM5_ESM.pdf]

nature portfolio

Corresponding author(s): Osman Bakr and Makhsud I. Saidaminov

Solar Cells Reporting Summary

Nature Portfolio wishes to improve the reproducibility of the work that we publish. This form is intended for publication with all accepted papers reporting the characterization of photovoltaic devices and provides structure for consistency and transparency in reporting. Some list items might not apply to an individual manuscript, but all fields must be completed for clarity.

For further information on Nature Research policies, including our data availability policy, see Authors & Referees.

Experimental design

Please check the following details are reported in the manuscript, and provide a brief description or explanation where applicable.

1. Dimensions

Area of the tested solar cells

☒ Yes
☐ No

Since each crystal varied in area, the active area also differed between cells. As described in the 'Methods' section, the glass side of each single-crystal solar cell was outlined using black opaque tape to form a photomask, which defined the active area. The active area for each crystal was then measured using an optical microscope. The active area varied from 0.7 mm2 to 3.6 mm2.

Explain why this information is not reported/not relevant.

Method used to determine the device area

☒ Yes
☐ No

'Device fabrication' sub-section under 'Materials' section of the main manuscript. The device area was measured using a calibrated microscope.

Explain why this information is not reported/not relevant.

2. Current-voltage characterization

Current density-voltage (J-V) plots in both forward and backward direction

☒ Yes
☐ No

Supplementary Figure 3a

Voltage scan conditions

☒ Yes
☐ No

J-V scan rate was 100 mV/s for both forward and reverse scan.

Explain why this information is not reported/not relevant.

Test environment

☒ Yes
☐ No

All cells were measured in a nitrogen filled glovebox.

Explain why this information is not reported/not relevant.

Protocol for preconditioning of the device before its characterization

☐ Yes
☒ No

Provide a description of the protocol.

No preconditioning was done.

Stability of the J-V characteristic

☐ Yes
☒ No

Provide a description of the method used. The stability of the J-V characteristic can be verified with time evolution of the maximum power point or with the photocurrent at maximum power point; see ref. 5 for details.

Stability of J-V not relevant to our study.

3. Hysteresis or any other unusual behaviour

Description of the unusual behaviour observed during the characterization

☒ Yes
☐ No

Hysteresis was observed (Supplementary Figure 3a)

Explain why this information is not reported/not relevant.

Related experimental data

☒ Yes
☐ No

Supplementary Figure 3a

Explain why this information is not reported/not relevant.

4. Efficiency

External quantum efficiency (EQE) or incident photons to current efficiency (IPCE)

☒ Yes
☐ No

Supplementary Figure 3b

Explain why this information is not reported/not relevant.

A comparison between the integrated response under the standard reference spectrum and the response measure under the simulator

☒ Yes ☐ No

The integrated Jsc value obtained from EQE was consistent with the Jsc values obtained from J-V measurements (Supplementary Figure 3a and Supplementary Figure 3b).

Explain why this information is not reported/not relevant.

For tandem solar cells, the bias illumination and bias voltage used for each subcell

☐ Yes ☒ No

Provide a description of the measurement conditions.

No tandem solar cell were reported in this paper.

5. Calibration

Light source and reference cell or sensor used for the characterization

☒ Yes ☐ No

'Material and device characterization' sub-section under 'Methods' section of the main manuscript.

Explain why this information is not reported/not relevant.

Confirmation that the reference cell was calibrated and certified

☒ Yes ☐ No

The Si reference cell was calibrated and certified by Newport.

Explain why this information is not reported/not relevant.

Calculation of spectral mismatch between the reference cell and the devices under test

☐ Yes ☒ No

Provide a value of the spectral mismatch and/or a description of how it has been taken into account in the measurements.

The light spectrum used for measurements matches well with that of the reference silicon cell. Therefore, we did not determine the spectral mismatch factor between the reference cell and the tested devices.

6. Mask/aperture

Size of the mask/aperture used during testing

☒ Yes ☐ No

Since each crystal varied in area, the active area also differed between cells. As described in the 'Methods' section, the glass side of each single-crystal solar cell was outlined using black opaque tape to form a photomask, which defined the active area. The active area for each crystal was then measured using an optical microscope. The active area varied from 0.7 mm2 to 3.6 mm2.

Explain why this information is not reported/not relevant.

Variation of the measured short-circuit current density with the mask/aperture area

☐ Yes ☒ No

Report the difference in the short-circuit current density values measured with the mask and aperture area.

This paper does not focus on device performance as a function of aperture area.

7. Performance certification

Identity of the independent certification laboratory that confirmed the photovoltaic performance

☐ Yes ☒ No

Identify the independent certification laboratory.

No certification was done.

A copy of any certificate(s)

☐ Yes ☒ No

Certificate copies should be provided in the Supplementary information. Please state the supplementary item number.

No certification was done.

8. Statistics

Number of solar cells tested

☒ Yes ☐ No

Caption of Figure 2, Supplementary Figure 4. We measured 10 solar cells for each variation.

Explain why this information is not reported/not relevant.

Statistical analysis of the device performance

☒ Yes ☐ No

Figure 2c, Figure 2d, Figure 2e, Figure 2f, Supplementary Figure 4c, Supplementary Figure 4d, Supplementary Figure 4e,Supplementary Figure 4f.

Explain why this information is not reported/not relevant.

9. Long-term stability analysis

Type of analysis, bias conditions and environmental conditions

☒ Yes ☐ No

We measured the shelf-stability of solar cells stored in nitrogen-glovebox (Supplementary Figure 13) and in ambient conditions (Supplementary Figure 14). We also evaluated the operational stability of solar cells by doing maximum power point tracking (MPPT) measurements (Supplementary Figure 15).

Explain why this information is not reported/not relevant.
